# Genetic association of *PROC* variants with pulmonary embolism in Northern Chinese Han population

**DOI:** 10.1186/s40064-016-1801-9

**Published:** 2016-02-24

**Authors:** Zengliang Wang, Tianhe Wang, Jianyong Chang, Hua Li, Chengdong Wang, Yongyong Li, Xuhe Lang, Shimei Jing, Guoqing Zhang, Yuting Wang

**Affiliations:** Department of Thorax, Anqiu People’s Hospital, Weifang, 262100 China; Department of Brain EMG, Anqiu People’s Hospital, Weifang, 262100 China; Department of Neurosurgery, Weifang People’s Hospital, Weifang, 261021 China; Department of Neurology, Anqiu People’s Hospital, Weifang, 262100 China; Key Laboratory of Weifang Brain Hospital, Weifang People’s Hospital, Weifang, 261021 China; Department of Surgery, Anqiu Municipal Hospital, Weifang, 262100 China; Department of Nephrology, Anqiu People’s Hospital, Weifang, 262100 China; Department of Neurosurgery, People’s Hospital of Weifang High Tech Industry Development Zone, Weifang, 261041 China

**Keywords:** Pulmonary embolism, *PROC* (protein C gene), Single nucleotide polymorphism

## Abstract

To evaluate SNPs (single nucleotide polymorphism) in *PROC* (protein C gene) associated with pulmonary embolism (PE) susceptibility in North Chinese Han population. A case-control study design was used, and patients with PE and healthy participants were enrolled from the Emerging Department of the several hospitals in Weifang, Shandong, China. SNPs in *PROC* were genotyped using Mass ARRAY system. The allele frequency of rs199469469 was significantly different between PE patients and the control [OR (95 % CI) = 5.00 (1.66–15.12), *P* = 0.004], and the difference remained significantly after controlling for age and gender [OR (95 % CI) = 5.34 (1.47–19.39), *P* = 0.011). The G(del)G in the haplotype includes rs1799809|rs199469469|rs2069928 was of a significantly difference (*P* = 0.016) among PE patients and the controls, and remained significant (*P* = 0.015) after adjustment for age and sex. Our study reports that *PROC* SNPs (rs199469469) might be associated with PE susceptibility, with the G allele of rs199469469 serving as the protective factors for incidence of PE. These findings may contribute to the understanding and primary prevention of PE.

## Background

The major thrombotic medical disorders include venous thromboembolism (VTE), a multifactorial disorder consisting of deep venous thrombosis (DVT) and pulmonary embolism (PE). PE is one manifestation of venous thromboembolism (VTE) and is a frequent, recurrent and potentially fatal disease (Goldhaber and Bounameaux 2012; Goldhaber 2012). PE contributes to 5–10 % of deaths in hospitalized patients and VTE is a leading preventable cause of in-hospitalized death (Alikhan et al. 2004; Cohen et al. 2008). In USA, the incidence of all DVT/PE events is 300,000–600,000 cases per year (approximately 1–2 per 1000 persons per year) and the mortality rates of all DVT/PE events is 60,000–100,000 cases per year (Beckman et al. 2010). However, it is difficult to estimate accurate mortality rates of the PE, because of the presence of related diseases and the large proportion of undiagnosed PE (Laack and Goyal 2004).With the improvement of its diagnosis and the development of access to healthcare, VT in Asian populations is now thought to be rising (Roberts et al. 2009; Zakai and McClure 2011).

PE is caused by both genetic and environmental factors, among which genetic factors account for up to 60 % of risk (Souto et al. 2000). Studies have shown that single nucleotide polymorphisms (SNPs) in genes (protein C gene, protein S gene and antithrombin gene) may contribute to the susceptibility to PE (Roberts et al. 2009; Suehisa et al. 2001). Protein C (*PROC*, or *PC*) is a vitamin K-dependent serine protease zymogen, which is an inactive zymogen and can be stimulated by the thrombin–thrombomodulin (TM)—endothelial protein C receptor complex on endothelial cell surfaces. TM-bound thrombin cleaves the 158–169 activation peptide of PC and generates activated PC (APC) (Wildhagen et al 2011). APC is a key component of the anticoagulation system. APC inhibits the coagulation pathway by proteolysis of coagulation factor Va (the activated form of coagulation factor V) and coagulation factor VIIIa (Cramer and Gale 2011). The decreasing of APC level is an independent risk factor for both venous and arterial thrombosis (Soare and Popa 2010).

The human *PROC* is located on 2q13–q14 and comprises 9 exons. Up to date more than 200 mutations were identified in the *PROC* gene (D’Ursi et al 2007). Most studies were conducted in Western populations, and few in the Asian population (Gandrille et al. 1995; Miyata et al. 1998; Reitsma et al. 1991; Shen et al. 1997). These studies show that the mutation pattern of *PROC* is of significant ethnic differences, thus, some mutations (Arg230Cys, Arg178Trp, Gln132X, Val297Met and Pro168Leu) are common in the Caucasian population, while the others (Phe139Val/ rs199469470, Arg169Trp/ rs759316085, Val297Met, Met364Ile, and G8857del) are observed in the Japanese population. So far, few studies have been published to investigate the association between *PROC* variants with incidence of PE in mainland China.

In this study, we investigated the association between *PROC* variants and incidence of PE in a case-control study of North China Han population.

## Results

**Fig. 1:**
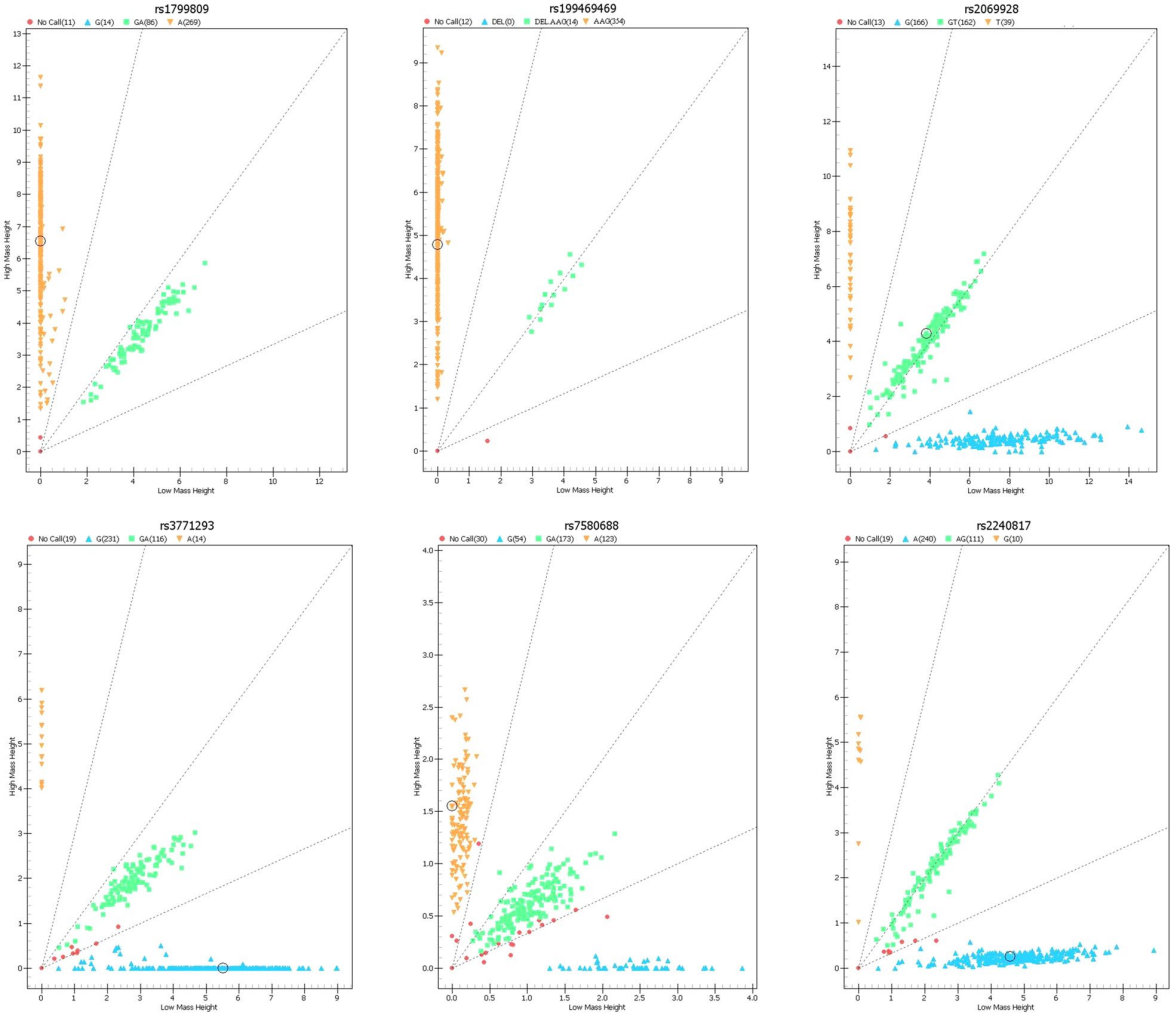
Genotyping of six SNPs in *PROC* gene

In total, 101 cases of PE (61 males and 40 females; with median age 63 year-old, ranged 24–85), and 279 healthy controls (187 males and 92 females; with median age 65 year-old, ranged 48–87 year-old) were finally included in the study. Six polymorphisms in the *PROC* gene were genotyped through Mass ARRAY system, with success rate of 99.7, 96.8, 96.6, 92.1, 97.6 and 97.6% for rs1799809, rs199469469, rs2069928, rs7580688, rs2240817 and rs3771293, respectively (Fig. 1).

All the six polymorphisms in cases and controls were both confirmed to Hardy–Weinberg equilibrium (Table 1). Table 1 showed that the “del” allele of rs199469469 (AAG/del) was of allele difference between the case and the control (OR = 5.00, 95 % CI 1.66–15.12, *P* = 0.004), and the association remained significant after adjustment for age and sex (OR = 5.34, 95 % CI 1.47–19.39, *P* = 0.011). For the other 5 SNPs, no statistical significances were observed (Table 1). rs199469469 was associated with PE in genotype and dominant model, but not in recessive model (Table 2).

**Table 1:** The allele frequency of SNPs in PROC in patients with PE and the controls

SNPs	Allele	Hardy–Weinberg equilibrium				Minor allele frequency (%)		Univariate analysis		Multivariate analysis^a^	
		The cases		The controls		Case	Control	OR (95% CI)	*P*	OR (95% CI)	*P*
		GENO	*P*	GENO	*P*						
rs1799809	A>G	5/25/71	0.170	9/61/198	0.140	0.173	0.147	1.212 (0.784–1.874)	0.424	1.131 (0.714–1.791)	0.599
rs199469469	AAG>del	0/9/91	1.000	0/5/263	1.000	0.045	0.009	5.004 (1.656–15.120)	0.004	5.339 (1.470–19.390)	0.011
rs2069928	G>T	11/45/45	1.000	28/117/121	1.000	0.332	0.325	1.030 (0.730–1.453)	0.861	1.102 (0.759–1.601)	0.609
rs7580688	A>G	16/54/29	0.310	38/119/94	1.000	0.434	0.388	1.209 (0.866–1.687)	0.267	1.270 (0.867–1.859)	0.219
rs2240817	A>G	4/28/69	0.510	6/83/171	0.400	0.178	0.183	0.970 (0.635–1.482)	0.915	1.187 (0.742–1.899)	0.476
rs3771293	A>G	4/37/60	0.770	10/79/171	0.840	0.223	0.190	1.219 (0.819–1.813)	0.351	1.155 (0.739–1.805)	0.528

*SNPs* single nucleotide polymorphisms, *CI* confidence interval, *OR* odds ratio

^a^Adjusted for sex and age

**Table 2:** the association between SNP rs199469469 with PE in three genetic models

Model	Case	Control	*P*
GENO	0/9/91	0/5/263	0.003
DOM	9/91	5/263	0.003
REC	0/100	0/268	1.000

The frequencies of Haplotype were evaluated using the Chi-square test, and Logistic regression analysis was performed to adjust for age and gender. The results showed that Haplotype G(del)G frequency (rs1799809|rs199469469|rs2069928) between the case and the control (*P* = 0.016) was of significant difference, and remained significant (*P* = 0.015) after adjustment for age and sex (Table 3).

**Table 3:** The haplotype distribution of PROC (rs1799809|rs199469469|rs2069928) in the cases and the controls

Haplotype	F-Affected	F-Control	Chi-square	*P*	*P*_*adj*_
G(AAG)T	0.027	0.030	0.049	0.824	0.922
A(AAG)T	0.310	0.296	0.130	0.718	0.630
G(Del)G	0.030	0.007	5.790	0.016	0.015
G(AAG)G	0.119	0.111	0.107	0.743	0.958
A(AAG)G	0.514	0.556	1.037	0.309	0.209

*P*_*adj*_ adjust for age and gender

We also described the allele frequency data in the present study as well as 1000 genome project. Except for rs199469469, the allele frequency of other 5 SNPs are comparable among the present study and these in 1000 Genome of different ethnic groups (Table 4). This paper confirms data reported by Tang et al. (2012b) who identified that the rs199469469 in PROC was associated with both decreased protein C anticoagulation activity and an increased risk of thrombosis in Chinese subjects of South China, whereas no allele frequency of rs199469469 were reported in 1000 Genome or other populations. Therefore, the “del” allele of rs199469469 could be considered typical of Chinese people, and should be validated in large cohort of different ethnic groups.

**Table 4:** The minor allele frequency of selected SNPs in the present study and 1000 genome

SNPs	Minor allele	The present study		1000 genome				
		Case	Control	CHB	CHS	JPT	CEU	YRI
rs1799809	G	0.173	0.147	0.175	0.152	0.139	0.409	0.806
rs199469469	del	0.045	0.009	–	–	–	–	–
rs2069928	T	0.332	0.325	0.374	0.295	0.255	0.248	0.088
rs7580688	G	0.434	0.388	0.369	0.371	0.260	0.313	0.588
rs2240817	G	0.178	0.183	0.151	0.133	0.130	0.152	0.120
rs3771293	G	0.223	0.190	0.199	0.219	0.130	0.152	0.278

*CHB* Han Chinese Beijing; *CHS* Southern Han Chinese; *JPT* Japanese in Tokyo; *YRI* Yoruba in Ibadan, Nigeria; *CEU* Utah residences with Northern and Western Ancestry

## Discussion

In this study, we investigated the association between *PROC* variants and the development of PE, and revealed that the rs199469469 (also designated as c.574_576del or p.Lys150del, located in exon 7 of the *PROC* gene) predispose people to PE. The case-control study showed that variant confers an approximately 5.00-fold increased risk of PE in the north Chinese population, and the results persist after adjustment for age and sex (OR = 5.34, *P* = 0.011).

*PROC*, with a single chain and synthesized by hepatocytes, is composed of a short activation peptide, a serine protease domain, carboxy-glutamic acid residue (Gla) domain, two epidermal growth factor (EGF)-like domains (Fisher et al. 1994; Perera et al. 2000). The deletion of lysine in *PROC* (rs19469469) can caused conformational changes in the protease domain (Chen et al. 2008), resulting in the elimination of a positive charge (Wildhagen et al. 2011). The most important is that the deletion is located at the ‘linking peptide (residues 137–157)’, which precedes the activation peptide and excised upon protein C activation. Residues Gly142–Leu155 are well conserved among species, and previously researches showed the region is important for anticoagulant activity (Mesters et al. 1993; Lu et al. 2013). Factor-V-Leiden mutation in the coding sequence of *F5* (Dahlbäck et al. 1993; Koster et al. 1993; Bertina et al. 1994)^,^ prothrombin-G20210A mutation in the 3′ UTR of *F2* and antithrombin-Cambridge-II mutation in *SERPINC1* are common genetic risk factors for VT in whites (Poort et al. 1996; Corral et al. 2007). However, these polymorphisms are rare in Asians, including Chinese populations. Little is known about the genetic background of VT, and no common genetic risk factors have been identified in the Chinese population until recently. Tang et al (2012a) carried out the first study on the genetic background of PC deficiency in the Chinese population, and identified a common mutation in *PROC* (rs146922325, c.565C>T) in a family study (first-degree relatives bearing this variant had an 8.8-fold increased risk of venous thrombosis), which has been further verified by our case-control study (the mutant allele conferred a high predisposition to venous thrombosis (adjusted OR = 7.34, 95 % CI 3.61–14.94). Tang et al (2012b, 2013) further identified that rs199469469 in *PROC* was associated with both decreased protein C anticoagulant activity and an increased risk of VTE in Chinese (Hubei Province), with an odds ratio of 2.93, and 2.84 (Tang et al. 2012b, 2013). The studies by Tang et al (2012a, b, 2013) were performed in Southern China, and our study performed in Northern China confirmed that rs199469469 in *PROC* was associated with an increased risk of VTE in Chinese.

## Conclusion

We firstly showed that the “del” allele of rs199469469 in *PROC* is associated with the increased risk of PE in a Northern Chinese Han population and the finding was consistent with that in Southern Chinese. Further epidemiologic studies are required to determine this association in larger populations, and functional studies are needed to determine the effect of this mutation contributing to susceptibility to PE. These studies will be a benefit to early predication or primary prevention of PE.

## Methods

### Participants

This study was approved by the ethics committee of Weifang People’s Hospital. A total of 101 unrelated patients diagnosed with PE in 2011 and 2012 were recruited from the Emerging Department of the hospitals in North China. The diagnosis of PE met the criteria recommended by the European Society of Cardiology (ESC) published in 2008 (Torbicki et al. 2008), and the patients with PE for patent foramen ovale were not screened. The inclusion criteria were: (1) Chinese Han population; (2) > or = 18 year-old. While the exclusion criteria were: (1) patients with acute liver disease or nephrotic syndrome; (2) recurrent venous thrombosis or pulmonary embolism; (3) patients with personal or family history of venous thromboembolism or other blood disease; (4) patients with the special history of drug use; (5) patients with chronic diseases such as hypertension; (6) patients with insertion of pacemaker.

The controls were 279 ethnic matched healthy individuals recruited in physical examination. All the patients and the control have given their written consents to participate in the study.

### Selection of SNPs and genotyping

For the selection of candidate SNPs, we firstly downloaded all SNPs information in PROC region (including 5000 bp in the upstream and 5000 bp in the downstream) from Hapmap (Version 3, Release 2, Analysis Panel CHB), then we screened the SNPs with Minor Allele Frequency ≥0.10, and P ≥ 0.10 in Hardy–Weinberg Equilibrium test, finally we selected the tag SNPs (r^2^ ≥ 0.80) using Tagger function of Haploview (Barrett et al. 2005). Five SNPs near PROC (rs1799809, rs199469469, rs2069928, rs7580688, rs2240817) meet the criteria. rs199469469 was also added because it was associated with both decreased protein C anticoagulation activity and an increased risk of thrombosis in Chinese subjects of South China (Tang et al. 2012b).

Genomic DNA was extracted from whole-blood samples using QIAamp DNA Blood Mini Kit (Qiagen, German) according to the manual instructions. Genomic DNA samples were subsequently diluted to 25 ng/μl. SNPs were genotyped using Mass ARRAY system (Sequenom, Inc., SanDiego, CA). Sequenom Mass-ARRAY^®^ Assay Design 3.0 software (Sequenom, Inc., San Diego, CA, USA) was used to design Multiplexed SNP Mass-EXTEND assays (Gabriel et al. 2009). SNP genotyping was performed using the standard protocol recommended by the manufacturer with a Sequenom Mass-ARRAY^®^ RS1000 (Sequenom, Inc.). Sequenom Typer 4.0 software was used for data management and analyses (Thomas et al. 2007).

### Statistical analysis

The Hardy–Weinberg equilibrium (HWE) of each SNP was evaluated using the Chi-square test. Differences between groups were analyzed by a Student’s t test or Mann–Whitney U test depending on the distribution of the laboratory data for continuous variables. The associations between PE and specific *PROC* genotypes were estimated by computing the odds ratios (OR) and 95 % confidence intervals (95 % CI) from the Chi-square test or Fisher’s exact test. Logistic regression analysis was performed to adjust for conventional risk factors including age and gender. Two-sided significance level was 0.05. The statistical analyses were performed by SPSS 13.0 (SPSS Inc., Chicago, IL, USA).

## Abbreviations

SNP: single nucleotide polymorphism
*PROC*: protein C
PE: pulmonary embolism
VTE: venous thromboembolism
DVT: deep venous thrombosis
VTE: venous thromboembolism
TM: thrombin–thrombomodulin
APC: activated PC
Gla: glutamic acid residue
EGF: epidermal growth factor
ESC: European Society of Cardiology
HWE: the Hardy–Weinberg equilibrium
